# Supplementary material for: Differentiating between Bayesian parameter learning and structure learning based on behavioural and pupil measures
Source: PLoS One. 2023 Feb 16;18(2):e0270619. doi: 10.1371/journal.pone.0270619 (PMC9934335; doi:10.1371/journal.pone.0270619)
Supplement: S1 File — (DOCX) [file pone.0270619.s001.docx]

# **Supplementary materials**


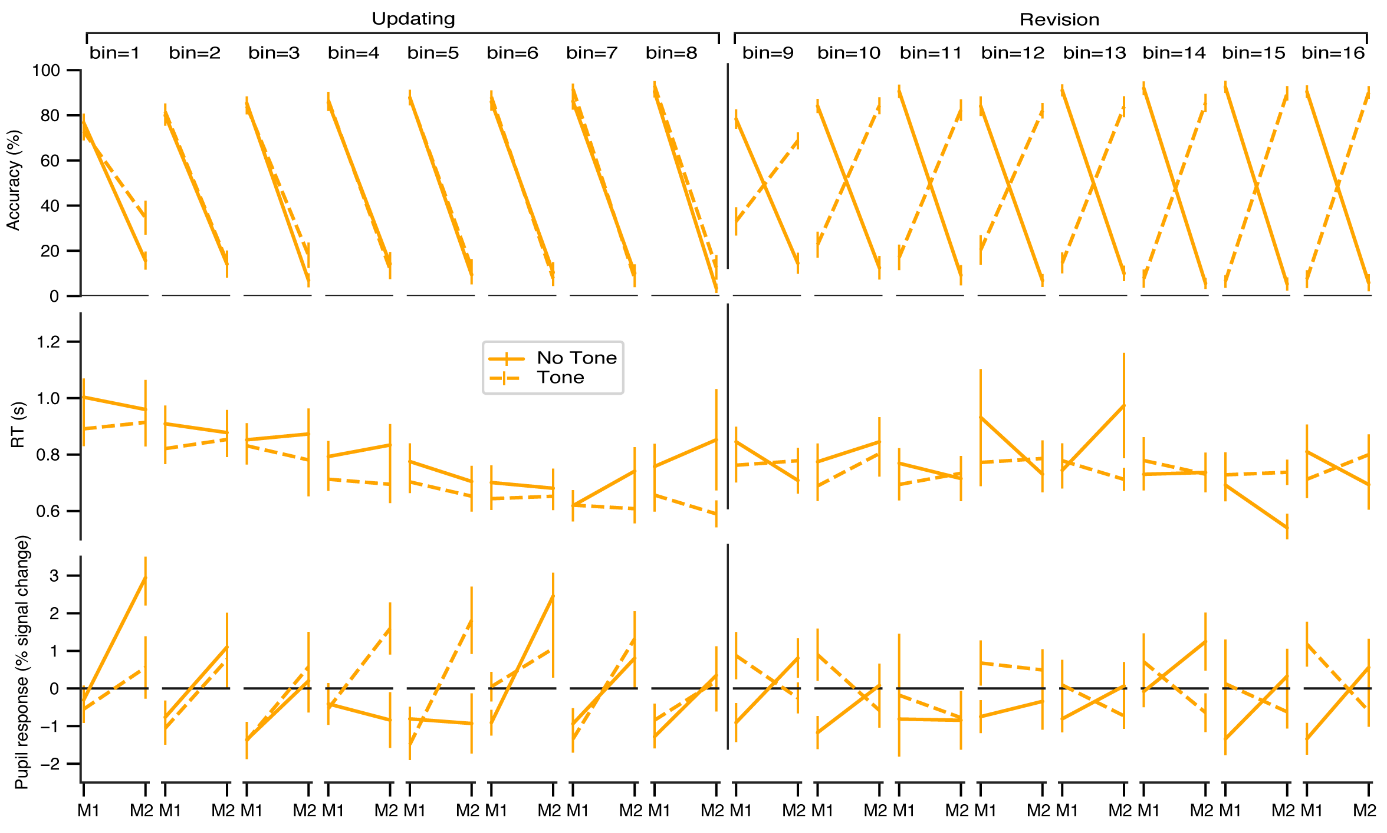


**Supplementary Figure 1. Cue-target prediction task results as a function of trial bin.** Prediction accuracy (top row), mean RT (middle row), and target-locked pupil dilation (bottom row) as a function of visual cue-target mapping condition (M1 vs. M2), the presence of the auditory cue (tone vs. no tone), and consecutive trial bin. Trial bins 1-8 correspond to the first phase, and trial bins 9-16 correspond to the second phase (25 trials per bin).

**Monotonic relationship between accuracy and target-locked pupil responses**

If pupil responses track how informative the target itself is relative to the predicted target orientation, we expected the difference in frequency conditions in accuracy to negatively scale with the difference in frequency conditions in information gained (i.e., target-locked pupil responses; see Figure 1C, compare the 20% vs. 80% conditions). In other words, the target-locked pupil responses were expected to “mirror” the learning trajectory obtained in the accuracy of the behavioural responses.

To test this, we computed the main effect of frequency in the tone trials as the difference between the M2 as compared with the M1 mapping conditions separately for the accuracy data and target-locked pupil responses (see also Supplementary Figure 1, tone trials). Note that we only investigated this relationship for the tone trials, because the frequencies of the M1 and M2 mappings only changed in both phases of the experiment when an auditory cue was present. Next, we performed a Spearman correlation between the frequency difference in accuracy and the frequency difference in the target-locked pupil responses across 16 trial bins (12-13 trials per bin) separately for each participant. An example participant can be seen in Supplementary Figure 2A. To help correct for skewedness, the correlation coefficients were converted using a Fisher *z*-transformation for statistical inference [1]. At the group level, the resulting z-transformed correlation coefficients were tested against zero with a Bayesian one-sample t-test.


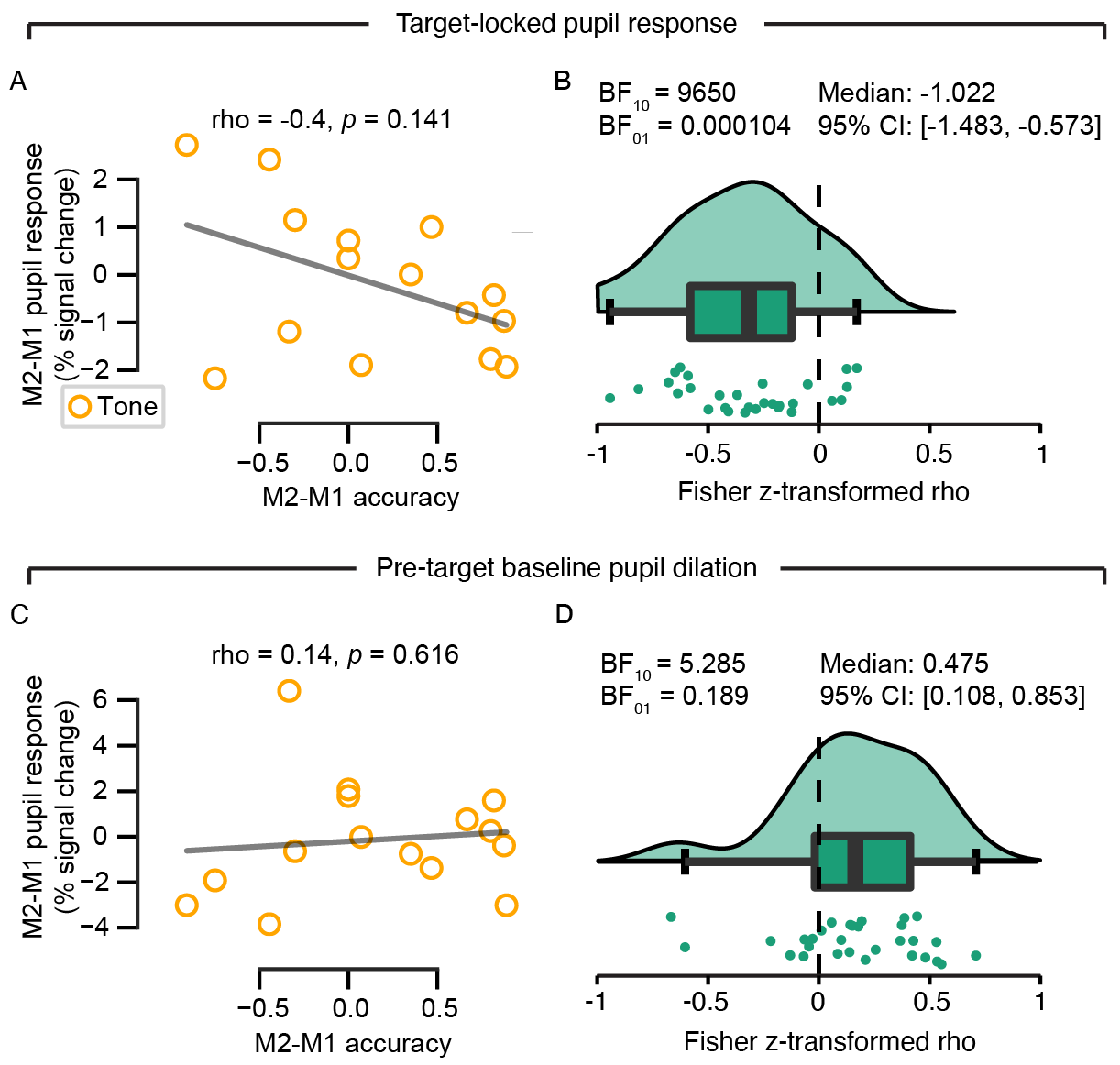


**Supplementary Figure 2. Monotonic relationship between behavioural and target-locked pupil responses.** (**A**) An example of one participant’s data is shown for the target-locked pupil responses (sub-1). The relationship between the frequency difference in accuracy and target-locked pupil responses across 16 trial bins (12-13 trials per bin). (**B**) The distribution of the Spearman correlation coefficients (Fisher *z*-transformed) at the group level for the target-locked pupil response. (**C**) An example of one participant’s data is shown for the pre-target baseline pupil dilation (sub-1). (**D**) The distribution of the Spearman correlation coefficients (Fisher *z*-transformed) at the group level for the pre-target baseline pupil dilation. Bayesian one-sample t-tests (against zero) were performed to evaluate the significance of the correlations.

Finally, we repeated the above analysis with the pre-target baseline pupil dilation in place of the target-locked pupil response to test whether the result was general for the pupil or specific to the target-locked pupil response ([2], [3], [4], [5], [6], [7], [8]). An example of the same participant can be seen in Supplementary Figure 2C.

At the group level, we obtained a Bayes factor of 9650 that suggests there was more evidence for the alternative hypothesis than for the null hypothesis of no correlation (Supplementary Figure 2B). An average negative correlation (*M* = -0.35, *SD* = 0.321) indicated that when participants had a larger difference between frequency conditions in accuracy, they also tended to have a smaller difference between frequency conditions in the target-locked pupil responses.

Furthermore, we confirmed that the negative scaling of the frequency effect in pupil dilation and accuracy was specific for the target-locked pupil responses (compare Supplementary Figure 2B with 2D). The results indicated that there is only anecdotal evidence to suggest that the difference between frequency conditions in the pre-target baseline pupil correlated (*M* = 0.16, *SD* = 0.32) with the frequency effect in behaviour (BF_10_ = 5). Finally, we confirmed that the two correlations of behaviour with i) the target-locked pupil response and ii) pre-target baseline pupil dilation differed at the group level (BF_10_ = 2934 in favour of the alternative hypothesis).

**Accuracy as a factor of interest for the target-locked pupil response**

The target-locked pupil dilation might reflect the difference in the frequency of the cue-target mapping conditions, but it also may reflect the *direction* of updating of current beliefs following novel sensory evidence indicating whether the outcome is better or worse than expected. For instance, a *correct* response on a *low frequency* (20%) trial may reflect a wrong button press on the part of the participant but may elicit a substantial amount of information gained due to the unlikely outcome.

We aimed to test this in a 4-way interaction between the factors: accuracy (error vs. correct), and cue-target mapping (M1 vs. M2), auditory cue (tone vs. no tone), and phase (first vs. second). We expected that the size of the two-way interaction term defined by the accuracy and cue-target mapping factors should change over time in accordance with the auditory-cue rules in the first and second phase.

We did not proceed with the above analysis due to too many missing cases across the 16 conditions determined by the 4-way interaction (*N* = 9 remaining in total). These missing cases were due to the rare occurrence of certain conditions, such as correct and low frequency trials. Therefore, we could not make any inference on the potential dynamics of this 4-way interaction in the current task design.

To increase statistical power, we collapsed across the cue-target mapping and auditory tone conditions and explored potential interactions between accuracy with the cue-target frequency and experimental phase (*N* = 22 remaining in total). We explored whether the target-locked pupil response would, on average, differentiate between the direction of updating current beliefs following the cue-target frequency conditions (i.e., a difference in the error and correct responses for each of the frequency conditions) and whether the interaction between accuracy and cue-target frequency would differ between the experimental phases. We performed a 3-way repeated measures ANOVA on the factors: accuracy (error vs. correct), cue-target frequency (80% vs. 20%), and experimental phase (first vs. second). The data are shown in Supplementary Figure 3.

**
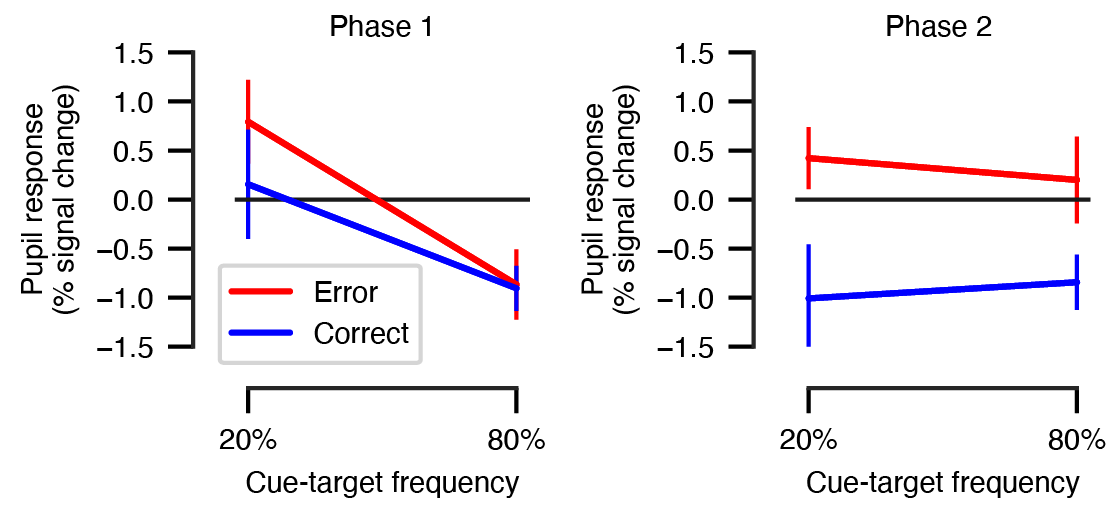
**

**Supplementary Figure 3. The target-locked pupil response as function of accuracy, cue-target frequency, and experimental phase.** Results of the 3-way repeated measures ANOVAs are given in Supplementary Table 1. Error bars, s.e.m. (*N* = 22).

The results of the 3-way ANOVA are given in Supplementary Table 1. Main effects of both accuracy and frequency were obtained, but these factors did not interact. As expected, errors elicited larger target-locked pupil responses as compared with correct trials (*M* = 0.94, *SE* = 0.31; see also Figure 3B for the time course of the responses), and low-frequency trials elicited larger pupil responses as compared with the high-frequency trials (*M* = 0.82, *SE* = 0.28). We note that the absence of an interaction effect may be partly due to the cue-target contingencies reversing on the tone trials (i.e., 80% -> 20% and 20% -> 80%) in the second phase of the experiment. In other words, this “flip” in the direction of expectancy of the tone trials between the two phases of the experiment may be adding noise to the averaged signal. In line with this, we found an interaction between frequency and phase, indicating that the pupil responses in the low-frequency condition were larger compared with the high-frequency condition in the first phase of the experiment (*t*(21) = 3.57, *p* = 0.005; *M* = 1.39, *SE* = 0.39), but not in the second phase (*t*(21) = 0.67, *p* = 0.507; *M* = 0.26, *SE* = 0.39).

**Supplementary Table 1. Results of the 3-way repeated measures ANOVAs on accuracy, frequency, and phase in the target-locked pupil response.** Factors of interest were accuracy (levels: error vs. correct), cue-target frequency (levels: 80% vs. 20%), and experimental phase (first vs. second). Pupil data were in percent signal change units. **p* < .05, ***p* < .01, ****p* < .001

|  |  |  | Pupil response | | |
| --- | --- | --- | --- | --- | --- |
| Effect |  |  | *F(1,21)* | *p* | *η²_G_* |
| Accuracy |  |  | 9.35 | < .006** | 0.04 |
| Frequency |  |  | 8.52 | 0.008** | 0.03 |
| Phase |  |  | 0.17 | 0.681 | < .01 |
| Accuracy * Frequency |  |  | 0.75 | 0.396 | < .01 |
| Accuracy * Phase |  |  | 1.80 | 0.194 | < .01 |
| Frequency * Phase |  |  | 4.47 | 0.047* | 0.02 |
| Accuracy * Frequency * Phase |  |  | 0.23 | 0.640 | < .01 |


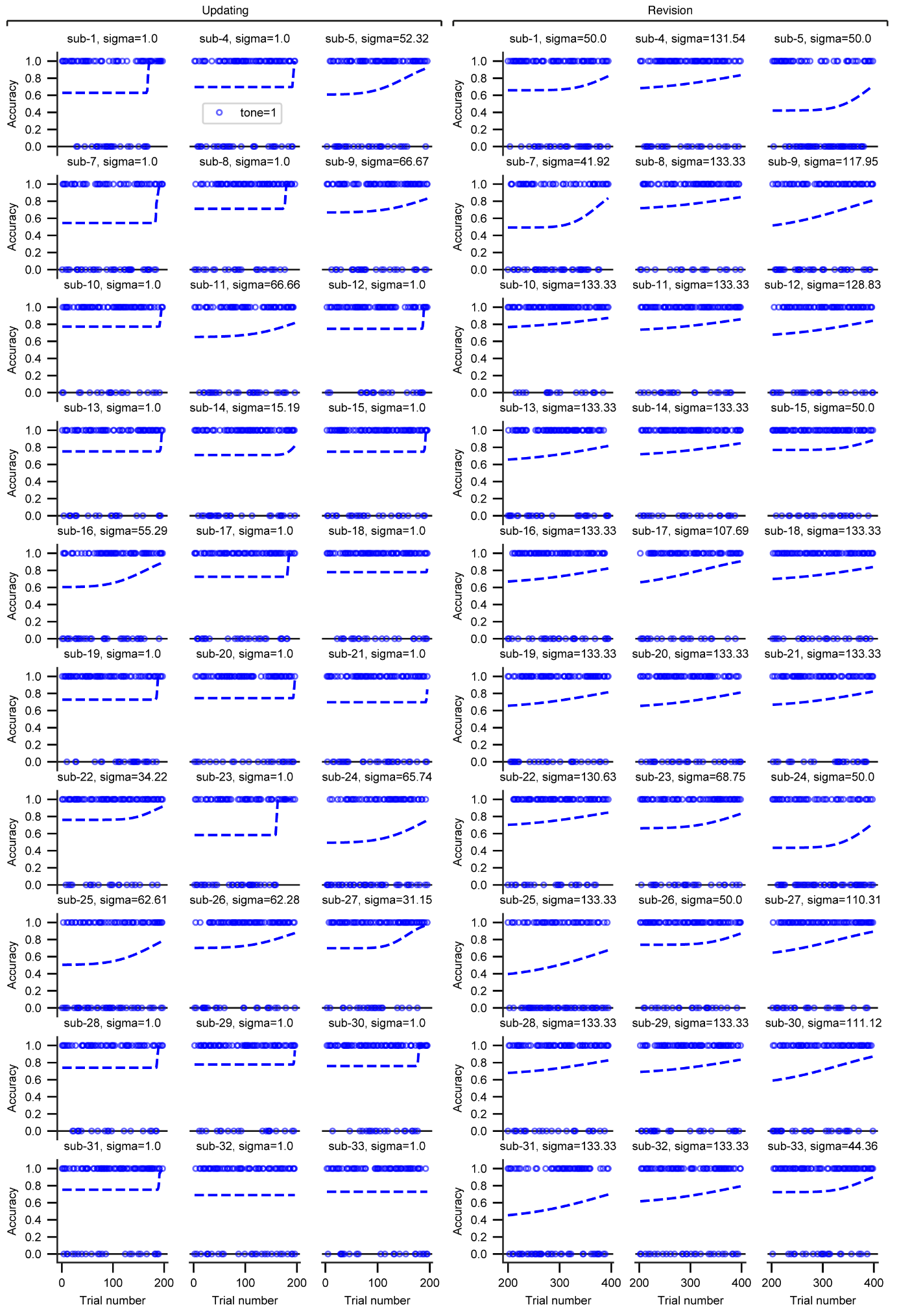


**Supplementary Figure 4. Psychometric curve fits for accuracy (single trial).** Individual psychometric curves were fit on the response accuracy data for the tone trials (auditory cue condition) separately for each phase (first vs. second).


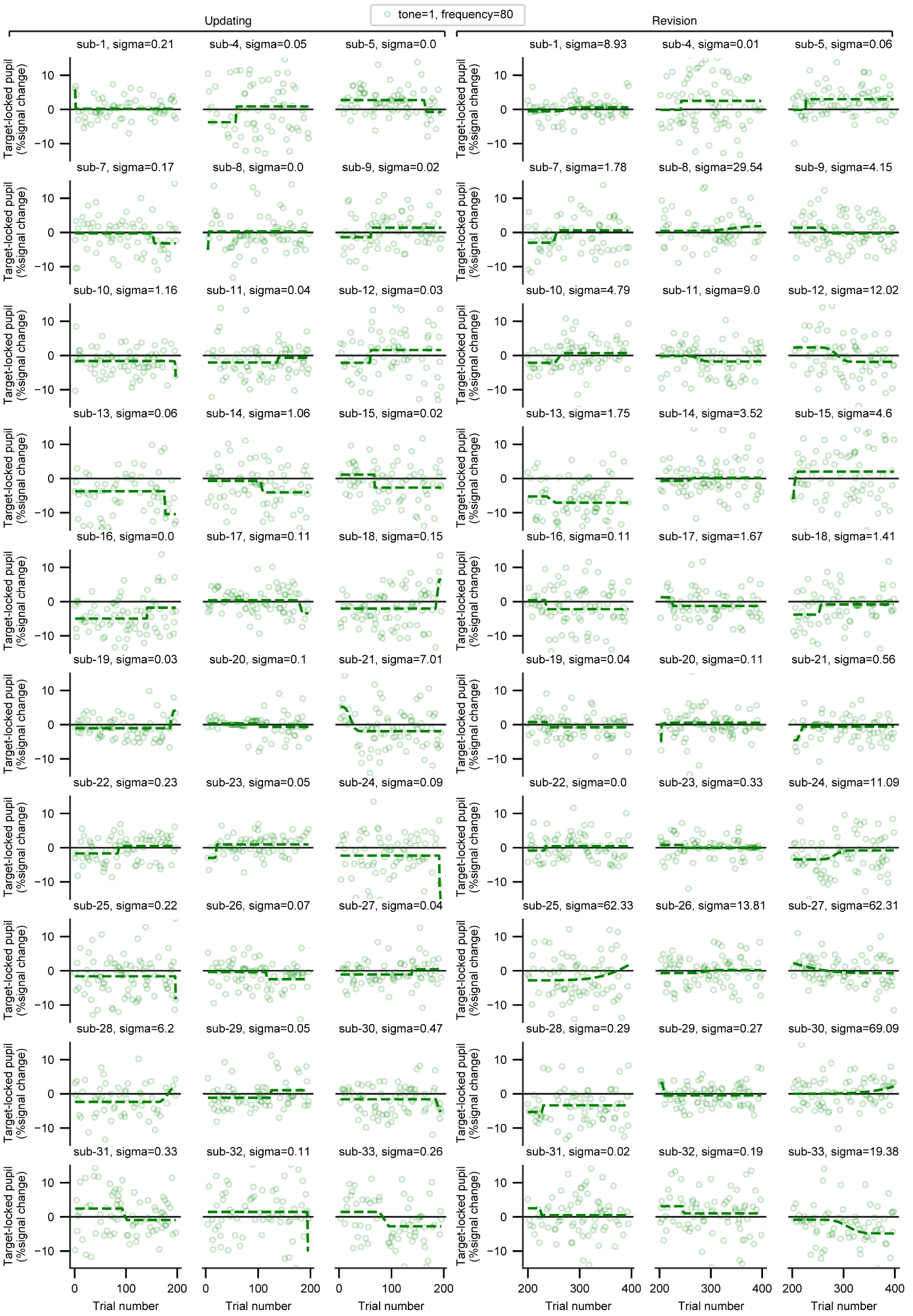


**Supplementary Figure 5. Curve fits for the target-locked pupil response (single-trial).** Individual curves were fit on the target-locked pupil response data for the high-frequency tone trials (i.e., the auditory cue and 80% frequency condition) separately for each phase (first vs. second).

**Supplementary Table 2. Descriptive statistics on free parameters from curve fits for the accuracy and pupil data.**

| **Accuracy** | | | | | | |  |  |
| --- | --- | --- | --- | --- | --- | --- | --- | --- |
|  | **μ** | | **σ** | | **a0** | |  |  |
|  | **First phase** | **Second phase** | **First phase** | **Second phase** | **First phase** | **Second phase** |  |  |
| Valid | 30 | 30 | 30 | 30 | 30 | 30 |  |  |
| Missing | 0 | 0 | 0 | 0 | 0 | 0 |  |  |
| Median | 191.29 | 400 | 1 | 132.437 | 0.711 | 0.649 |  |  |
| Mean | 187.996 | 389.102 | 17.737 | 108.104 | 0.69 | 0.617 |  |  |
| Std. Deviation | 13.053 | 22.626 | 26.1 | 36.207 | 0.08 | 0.108 |  |  |
| Minimum | 156.964 | 323.083 | 1 | 41.918 | 0.492 | 0.351 |  |  |
| Maximum | 200 | 400 | 66.667 | 133.333 | 0.779 | 0.77 |  |  |
| **Pupil response** | | | | | | | | |
|  | **μ** | | **σ** | | **a0** | | **G** | |
|  | **First phase** | **Second phase** | **First phase** | **Second phase** | **First phase** | **Second phase** | **First phase** | **Second phase** |
| Valid | 30 | 30 | 30 | 30 | 30 | 30 | 30 | 30 |
| Missing | 0 | 0 | 0 | 0 | 0 | 0 | 0 | 0 |
| Median | 130.827 | 246.795 | 0.099 | 1.766 | -1.269 | -0.365 | -2.522 | 1.036 |
| Mean | 122.354 | 262.328 | 0.612 | 10.772 | 0.767 | -1.99 | -4.136 | 2.397 |
| Std. Deviation | 63.868 | 60.348 | 1.656 | 19.487 | 9.846 | 8.267 | 11.938 | 9.307 |
| Minimum | 0.747 | 186.918 | 1.369e -5 | 8.487e -4 | -5.082 | -43.248 | -51.324 | -7.209 |
| Maximum | 198.993 | 457.277 | 7.008 | 69.092 | 51.521 | 6.503 | 8.48 | 45.274 |

**References**

1. Myers, L., & Sirois, M. J. (2006). Spearman Correlation Coefficients, Differences between. In *Encyclopedia of Statistical Sciences*. https://doi.org/10.1002/0471667196.ess5050.pub2
2. Gilzenrat, M. S., Nieuwenhuis, S., Jepma, M., & Cohen, J. D. (2010). Pupil diameter tracks changes in control state predicted by the adaptive gain theory of locus coeruleus function. *Cognitive, Affective, & Behavioral Neuroscience*, *10*(2), 252–269. https://doi.org/10.3758/CABN.10.2.252
3. Joshi, S., & Gold, J. I. (2020). Pupil Size as a Window on Neural Substrates of Cognition. *Trends in Cognitive Sciences*, *24*(6), 466–480. https://doi.org/10.1016/j.tics.2020.03.005
4. Larsen, R. S., & Waters, J. (2018). Neuromodulatory correlates of pupil dilation. *Frontiers in Neural Circuits*, *12*, 21.
5. Murphy, P. R., Robertson, I. H., Balsters, J. H., & O’Connell, R. G. (2011). Pupillometry and P3 index the locus coeruleus–noradrenergic arousal function in humans. *Psychophysiology*, *48*(11), 1532–1543. <https://doi.org/10.1111/j.1469-8986.2011.01226.x>
6. Murphy, P. R., O’Connell, R. G., O’Sullivan, M., Robertson, I. H., & Balsters, J. H. (2014). Pupil diameter covaries with BOLD activity in human locus coeruleus. *Human Brain Mapping*, *35*(8), 4140–4154. <https://doi.org/10.1002/hbm.22466>
7. Murphy, P. R., Vandekerckhove, J., & Nieuwenhuis, S. (2014). Pupil-Linked Arousal Determines Variability in Perceptual Decision Making. *PLOS Computational Biology*, *10*(9), e1003854. <https://doi.org/10.1371/journal.pcbi.1003854>
8. Vincent, P., Parr, T., Benrimoh, D., & Friston, K. J. (2019). With an eye on uncertainty: Modelling pupillary responses to environmental volatility. *PLOS Computational Biology*, *15*(7), e1007126. https://doi.org/10.1371/journal.pcbi.1007126
